# High-frequency rTMS in the treatment of depressive symptoms in schizophrenia: a neurophysiological profile of respondents and nonresponders

**DOI:** 10.1192/j.eurpsy.2022.1999

**Published:** 2022-09-01

**Authors:** V. Kaleda, A. Pomytkin, I. Lebedeva

**Affiliations:** 1 FSBSI «Mental Health Research Centre», Department Of Youth Psychiatry, Moscow, Russian Federation; 2 FSBSI «Mental Health Research Centre», Lab.neuroimaging And Multimodal Analysis, Moscow, Russian Federation

**Keywords:** TMS, schizophrénia, Depression, EEG

## Abstract

**Introduction:**

Depressive symptoms in schizophrenia have a high prevalence – up to 20-60 %, at the different illness stages. Non-pharmacological treatment, namely rTMS, seems like a promising approach that lacks side-effects typical for antidepressants. RTMS is widely applied in the treatment of depression, however the studies within schizophrenia domain are still rather few

**Objectives:**

The aim was to examine a potential of neurophysiological data for prediction of the effects of rTMS in the treatment of patients with schizophrenia with depressive symptoms

**Methods:**

20 male patients with schizophrenia (F20.004, F20.014, F20.414, ICD–10) were examined at the stage of incomplete remission with predominance of prolonged (more than 6 months) treatment resistant depressive symptoms. An examination (clinical and neurophysiological (oddball ERP and EEG) fragments)) was repeated twice - before and after a course of 10 Hz rTMS (left DLPC, 2000 pulses per session, 15 sessions).

**Results:**

Poor outcome was associated with initially higher coherence in alpha and lower - in beta1 EEG bands. The amplitudes of non-target N100 and mismatch negativity didn’t differ the groups of responders and nonresponders

**Conclusions:**

The disturbances within brain networks of beta1 and alpha generators merit attention as potential neurophysiological markers with predictive value in rTMS treatment of patients with schizophrenia with depressive symptoms.

**Disclosure:**

No significant relationships.

